# Effects of Ag Nanoparticles on Growth and Fat Body Proteins in Silkworms (*Bombyx mori*)

**DOI:** 10.1007/s12011-017-1001-7

**Published:** 2017-03-30

**Authors:** Xu Meng, Nouara Abdlli, Niannian Wang, Peng Lü, Zhichao Nie, Xin Dong, Shuang Lu, Keping Chen

**Affiliations:** Institute of Life Sciences, Jiangsu University, Zhenjiang, Jiangsu 212013 China

**Keywords:** AgNPs, *Bombyx mori*, Environmental risk, Fat body proteins, Growth

## Abstract

Ag nanoparticles (AgNPs), a widely used non-antibiotic, antibacterial material, have shown toxic and other potentially harmful effects in mammals. However, the deleterious effects of AgNPs on insects are still unknown. Here, we studied the effects of AgNPs on the model invertebrate organism *Bombyx mori*. After feeding silkworm larvae different concentrations of AgNPs, we evaluated the changes of *B. mori* body weights, survival rates, and proteomic differences. The results showed that low concentrations (<400 mg/L) of AgNPs promoted the growth and cocoon weights of *B. mori*. Although high concentrations (≥800 mg/L) of AgNPs also improved *B. mori* growth, they resulted in silkworm death. An analysis of fat body proteomic differences revealed 13 significant differences in fat body protein spots, nine of which exhibited significantly downregulated expression, while four showed significantly upregulated expression. Reverse transcription–polymerase chain reaction results showed that at an AgNP concentration of 1600 mg/L, the expression levels of seven proteins were similar to the transcription levels of their corresponding genes. Our results suggest that AgNPs lowered﻿ ﻿the resistance to oxidative stress, affected cell apoptosis, and induced cell necrosis by regulating related protein metabolism and metabolic pathways in *B. mori*.

## Introduction

Nanomaterials have very small sizes (1–100 nm) and some special physical and chemical properties. Ag nanoparticles (AgNPs) are some of the most novel and commercialized nanomaterials, and they have strong antibacterial activity. They are widely used in many fields, such as food packaging, medical devices, and cosmetics [1, 2]. However, several studies have implied that they are potentially hazardous [3, 4]. Currently, studies have demonstrated the potential impact of AgNPs on human health and the environment [3, 4]. Artificial nanomaterials have strong binding affinities for biopolymer molecules because of their lipophilic properties, coordination properties, and polarity effects both “in vivo” and in the environment, which have potentially adverse effects on human health and the environment [5–7]. Morones indicated that AgNPs not only exist on the cell membrane surface but can enter the cell interior [8]. The use of AgNPs in food storage may interfere with DNA replication and cause DNA mutations, which may potentially induce DNA denaturation [9]. Moreover, many nanomaterials can also enter the water, atmosphere, and soil, which is a huge potential risk to humans [10]. Studying the toxic effects of AgNPs on the model silkworm *Bombyx mori* can provide a useful reference for environmental monitoring.

In mammalian studies, nanomaterials entered different tissues and organs through the circulatory system, thereby endangering the safety of the host [11–14]. It was demonstrated that nanomaterials have adverse effects on tissues and organs, such as the brain, midgut, and reproductive organs [12, 13]. Nano-ZnO NPs, AgNPs, and nano-Ti_2_O NPs all had toxic effects on algae, zooplankton, and fish [15]. Furthermore, AgNPs showed potential toxicological and neurotoxicological effects in “vivo” and in “vitro” [11, 16, 17]. AgNPs induced slight liver injuries at doses of 125 mg/kg/day in rats in an oral exposure study [18]. These studies suggest that AgNPs have potent cytotoxic effects and may cause oxidative damage, inflammation, DNA damage, and cell apoptosis/necrosis [16, 17, 19].

Nanomaterials have potential risks to the environment, and their hazards are closely related to their concentration, morphology, migration, and transformation processes, as well as environmental conditions [20]. The toxicity of nanomaterials and their environmental risks have become a hot research topic. At present, studies of the toxic effects of AgNPs have mainly been conducted in mammals, while few studies have been conducted in invertebrates. Previous studies have reported that AgNPs can induce *Heliothis virescens* (tobacco budworm) and *Trichoplusia ni* (cabbage looper) developmental delay, reductions in adult weight and fecundity, and increased mortality in the predator [21]. *B. mori*, an important invertebrate model organism, exhibits relatively weak resistance to stress and disease, and it is especially sensitive to chemical pesticides, heavy metals, and other harmful substances [22]. The fat body plays an important physiological role in nutrient storage, metabolic detoxification, and immune regulation [23], and its function is similar to that of the mammalian liver [24, 25]. As such, it is a more sensitive model organism for monitoring environmental toxins. Studies found that AgNPs at the concentration of 100 ppm were able to produce lethal effects on pupation and adult development, with accumulative hazard in silkworm [26]. Here, we examined the effects of different concentrations of AgNPs on the growth of *B. mori*, and we also investigated the toxic effects of AgNPs by analyzing fat body proteomics in *B. mori*.

## Material and Methods

### Insect Strains

The larvae of *B. mori* (strain: Jingsong × Haoyue) were maintained in our laboratory and reared on mulberry (*Morus*) leaves under a 12-h light/12-h dark cycle. The larvae were fed three times per day.

### Chemicals

Silver nanoparticle (AgNP) powder was purchased from Suzhou Nord Derivatives Pharm-tech Co. Ltd. (Suzhou, China). Characterization of AgNPs (diameter 30 nm) and AgNP stock solution was synthesized as previously described [27]. The AgNPs were powdered using an ultrasonic technique for 20 min and mixed by mechanical vibration. To obtain the UV–vis spectrum of silver nanoparticles, powdered silver nanoparticles were dispersed in deionized water at 50 and 25 mg/L and scanned from 300 to 800 nm using a spectrophotometer (Synergy H4, Bio-Tek, USA). The size, shape, and dispersion of AgNPs were further confirmed by transmission electron microscopy (TEM, JEM-2100, JEOL, Japan).

### Treatments

Mulberry leaves were soaked in different concentrations of AgNPs. The soaked leaves were dried naturally at room temperature, and they were fed continuously three times per day to newly exuviated fourth- and fifth-instar larvae until molting. Control larvae were fed mulberry leaves soaked in water. All the larvae were maintained at 25 ± 0.5 °C and a relative humidity of 70–75%. Each treatment was replicated three times with 30 larvae. Furthermore, the fourth-instar silkworms were divided into two classes or seven groups. Class 1 received low concentrations of AgNPs, and it contained four treatment groups (double-distilled (dd)H_2_O and 100, 200, and 400 mg/L AgNPs). Class 2 received high concentrations of AgNPs, and it contained three treatment groups (800, 1600, and 3200 mg/L AgNPs). An analytical balance was used to measure the weights of the silkworms, and each value is the mean of three replicates.

### Protein Sample Preparation

Twenty silkworms were selected randomly for fat body extraction, and proteins were extracted with phenol. The silkworm fat body from the control (ddH_2_O) and treatment (AgNPs) groups was ground in liquid nitrogen with homogenization buffer (20 mM Tris–HCl, pH 7.5, 250 mM sucrose, 10 mM ethylenediaminetetraacetic acid, 1 mM phenylmethylsulfonyl fluoride, 1 mM beta-mercaptoethanol, and 1% (*v*/*v*) Triton X-100), as described by Cilia et al. [28]. Then, the mixture was vortexed for 30 min and centrifuged at 21000×*g* for 20 min. The supernatant was added to an equal volume of Tris-saturated phenol to precipitate the proteins. The phenol layer containing the proteins was collected, incubated with a methanol solution (containing 100 mM ammonium acetate), and centrifuged at 21000×*g* for 20 min to pellet the proteins. The pellet was washed with cold acetone (containing 1 mM dithiothreitol (DTT)); lyophilized, dissolved in a solution containing 7 M urea, 2 M thiourea, 4% (*w*/*v*) CHAPS, and 1% (*w*/*v*) DTT; and centrifuged at 21000×*g* for 20 min. The supernatant, as the sample of total fat body proteins, was pooled and stored at −80 °C for later use. The protein concentration was determined using the RC DC™ Kit (Bio-Rad, Hercules, CA, USA).

### Two-Dimensional Electrophoresis

Two-dimensional electrophoresis was performed with a 17-cm (linear, pH 4–7) immobilized pH gradient (IPG gel) strip (Bio-Rad), as described by Liang et al. [29]. Total fat body proteins (3 mg) were loaded onto the IPG strip for 12 h, and isoelectric focusing was performed at 20 °C with a voltage gradient of 100 V for 1 h, 300 V for 1 h, 1000 V for 1 h, 8000 V for 1 h, and 10,000 V for 40,000 Vh, and then, it was continued at 500 V. The IPG gel strip was equilibrated for 15 min with equilibration buffer (6 M urea, 0.375 M Tris–HCI, 20% (*v*/*v*) glycerol, 2% (*w*/*v*) sodium dodecyl sulfate (SDS), and 2% (*w*/*v*) DTT), and then, it was equilibrated for another 15 min in the same equilibration buffer without DTT, but containing 2.5% (*w*/*v*) iodoacetamide. The equilibrated strip was sealed on the top of a 12% SDS–polyacrylamide gel and subjected to electrophoresis. Proteins were visualized by staining with 0.1% Coomassie brilliant blue R-250, and they were scanned with a high-precision scanner (ImageScanner III, GE Healthcare Life Sciences, Pittsburgh, PA, USA) at a resolution of 300 dpi. Spot analysis was performed using ImageMaster (version 7.0, GE Healthcare Life Sciences). Triplicate experiments were conducted for each sample. The intensity ratio of the corresponding spots in different gels was calculated, and spots with ratio ≥2 and ANOVA ≤0.05 were defined as quantitatively different spots.

### RNA Extraction and Transcriptional Analysis

The fat bodies of the fifth-instar larvae in each group were dissected, immediately frozen in liquid N_2_, and stored at −80 °C for later use. Total RNA was extracted using TRIzol reagent (Invitrogen, Carlsbad, CA, USA). RNA was reverse transcribed from 3 μg of total RNA using Moloney Murine Leukemia Virus Reverse Transcriptase (Vazyme, Nanjing, China) according to the manufacturer’s instructions. NCBI Primer-BLAST (https://blast.ncbi.nlm.nih.gov/Blast.cgi) was used to design quantitative real-time polymerase chain reaction (qPCR) primers for important differentially expressed genes (Table 1); α-tubulin was used as the reference gene. qPCR was performed using a 7300 Fast System (Applied Biosystems, Foster City, CA, USA) with a SYBR Green Master Mix kit (Vazyme, Nanjing), according to the manufacturer’s instructions. The data were analyzed with the SDSS software package (Version 16.0, SPSS Inc., Chicago, IL, USA). All samples were measured independently three times.

**Table 1:** Primer sequences used in the qPCR

Gene name	Primer sequence (5′–3′)				Length of product (bp)
P1	F:	GTCCATCGACAGCGAGGAAT	R:	GGGCGTTCACATCCTCAGAA	167
P4	F:	GCTCCACTCACTGAAACCGA	R:	GGAACCACCGTTTTTGCTCC	203
P5	F:	ACGGTTGTTCAAGTGCCAGA	R:	AGGAGGGTGGATCCGAATGA	181
P6	F:	CCGGAGGCTCATCAGAAATCA	R:	TTCACATCACCCCCTTCTGC	164
P7	F:	GAGAGCGATCGGAAAAGGCT	R:	TAGAAGGGCTCATGCTGTCC	117
P8	F:	CCCCCGTGTTGGAAAACAAC	R:	ACGAAGAACATGACGTCGCT	190
P9	F:	ATGTGGGCATCAAATGTGCG	R:	AGCATGAGCATGACGTCCAA	206
P12	F:	GGAAAGCTGACATGGGGTGA	R:	AAGCCTTCACTTTGGGCTGT	106
P13	F:	CAATGCCTTAGCAGTGCGAC	R:	TCGGCTTTCGTCTTCAGGAG	239
α-Tubulin	F:	CTCCCTCCTCCATACCCT	R:	ATCAACTACCAGCCACCC	186

### Data Analysis

Statistical analyses were conducted using SPSS for Windows, Version 16.0. Data were expressed as the mean ± standard deviation (SD). One-way analysis of variance was conducted to compare the differences of the means among multi-group data. Dunnett’s test was performed when each dataset was compared with the control data. Statistical significance for all tests was judged at a probability level of 0.05 (*P* < 0.05).

## Results

### Characterization of AgNPs

**Fig. 1:**
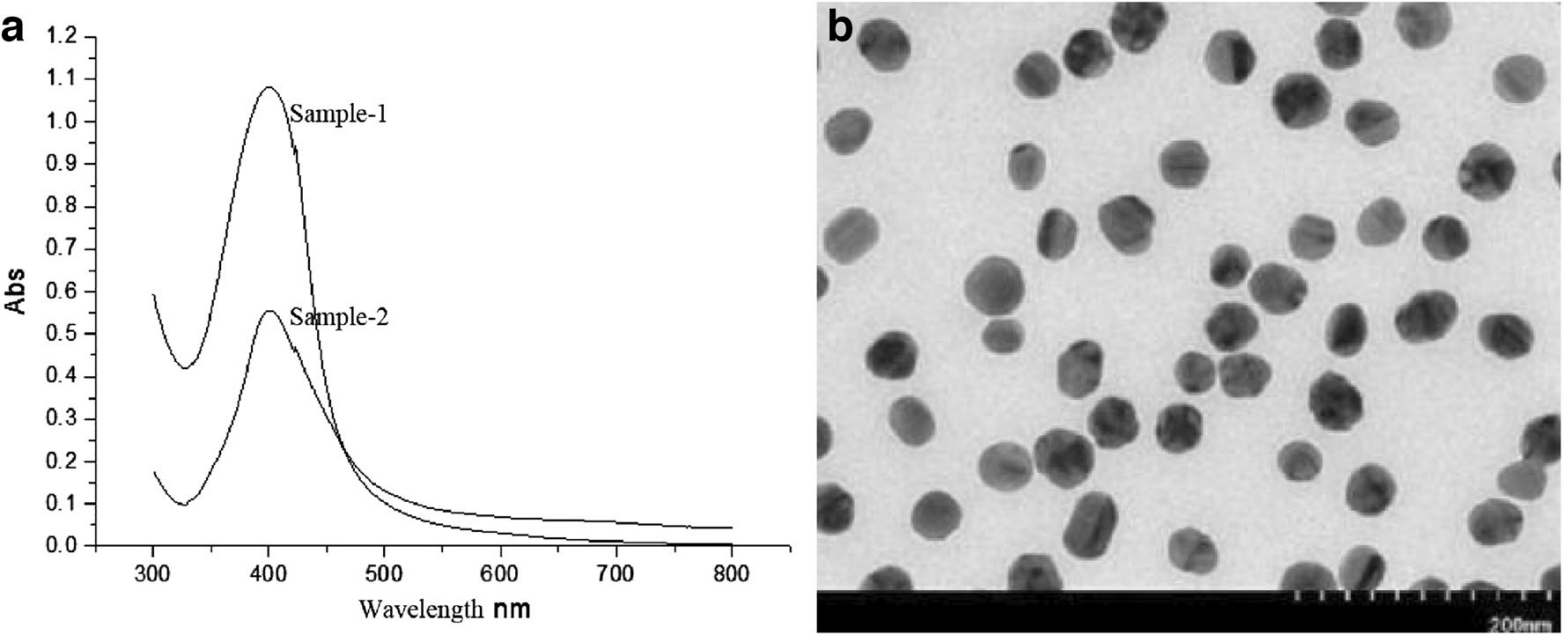
Characterization of AgNPs. **a** UV–visible absorption spectra of AgNPs powder dissolved in deionized water at 50 mg/L (sample 1) and 25 mg/L (sample 2). *Narrow peak* confirms the small size of the particles. **b** TEM image shows that the AgNPs exhibit the homogeneous distribution in size

The AgNPs employed in our study exhibited spherical characteristics with absorbance spectra at *λ*_max_ 400 nm (Fig. 1a). TEM images also substantiated the spherical silver nanoparticles with an approximate size of 30 nm (Fig. 1b). These data clearly indicated that experimental AgNPs exhibited a homogeneous dispersion in aqueous solutions.

### Effects of Feeding Different Concentrations of AgNPs on Silkworm Growth

**Fig. 2:**
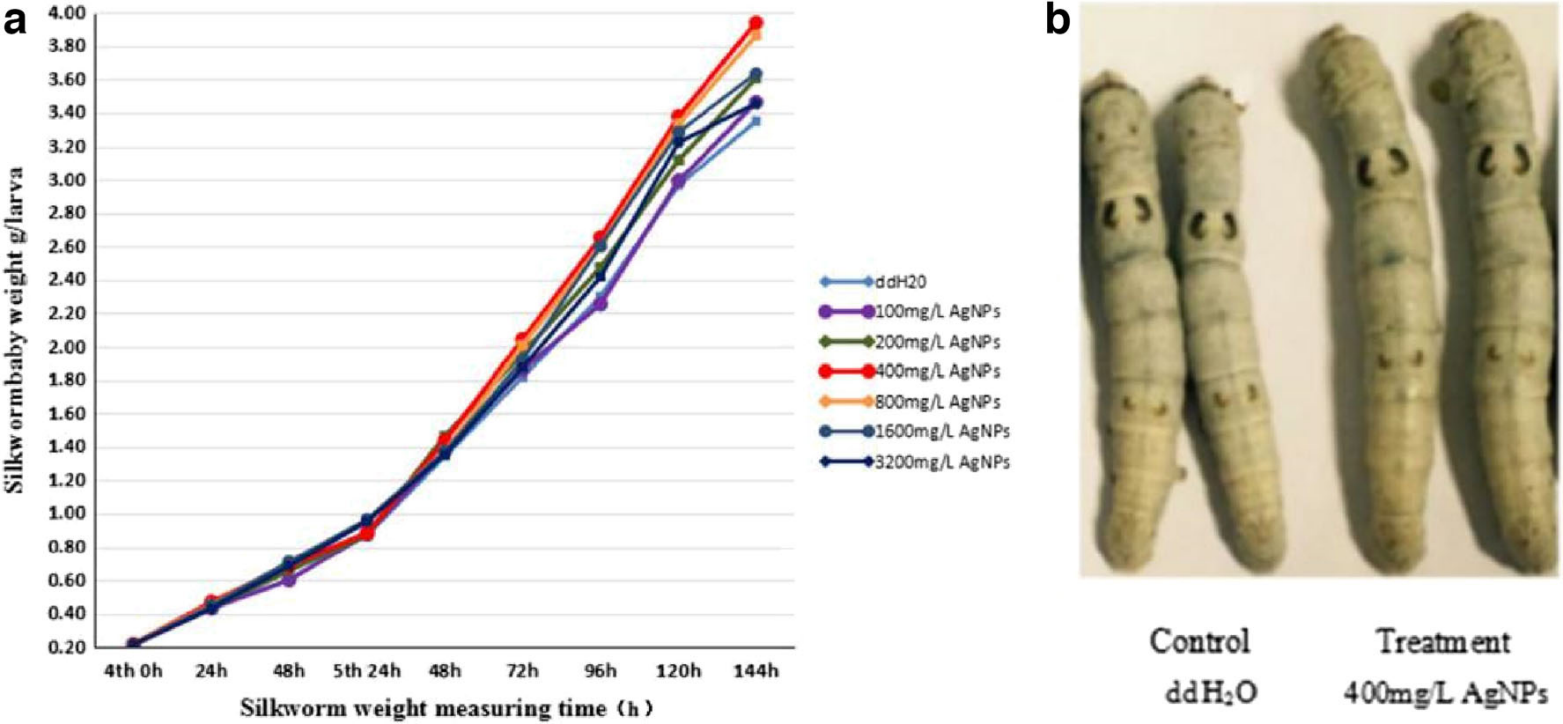
Effects of different concentrations of AgNPs on the body weights of silkworms. **a** Average weights of fourth- and fifth-instar silkworms from 0 to 48 and 24 to 144 h, respectively. **b** Morphological abnormalities of silkworms after feeding AgNPs. The body weights of the control (ddH_2_O) group differed from that of the treatment groups (400 mg/L AgNPs) during the fifth instar at 144 h

Silkworms were fed AgNPs from the fourth instar, and then, their body weights were measured. The results showed an increasing trend of the body weights with different concentrations of AgNPs (Fig. 2a and Table 2). The growth of *B. mori* that were fed <400 mg/L AgNPs did not change significantly during the fourth instar after 48 h, while their body weights increased slightly when fed >800 mg/L AgNPs. The body weights increased most significantly after the silkworms were fed 400 mg/L AgNPs for 144 h (Fig. 2b). The body weights of *B. mori* increased slowly at AgNP concentrations ≤200 mg/L, but the growth-promoting effect was diminished at higher (≥800 mg/L) AgNP concentrations (Table 2).

**Table 2:** Effects of feeding with different concentrations of AgNPs on silkworm baby weights

Ag NPs (mg/L)	4th silkworm baby weight (g)			5th silkworm baby weight (g)					
	0 h	24 h	48 h	24 h	48 h	72 h	96 h	120 h	144 h
0	0.217 ± 0.004 a	0.431 ± 0.032 a	0.656 ± 0.021 a	0.871 ± 0.041 a	1.346 ± 0.021 a	1.816 ± 0.048 a	2.301 ± 0.033 a	2.968 ± 0.167 a	3.352 ± 0.154 a
100	0.217 ± 0.002 a	0.433 ± 0.010 a	0.600 ± 0.017 b	0.877 ± 0.030 a	1.376 ± 0.026 a	1.863 ± 0.050 a	2.255 ± 0.069 a	2.994 ± 0.100 a	3.463 ± 0.221 a
200	0.218 ± 0.003 a	0.436 ± 0.011 a	0.657 ± 0.021 a	0.874 ± 0.017 a	1.465 ± 0.052 b	1.928 ± 0.092 a	2.475 ± 0.051 a	3.12 ± 0.100 a	3.607 ± 0.089 a
400	0.219 ± 0.015 a	0.469 ± 0.001 b	0.688 ± 0.017 a	0.885 ± 0.020 a	1.438 ± 0.050 b	2.043 ± 0.135 b	2.653 ± 0.062 b	3.379 ± 0.030 b	3.94 ± 0.089 b
800	0.218 ± 0.001 a	0.453 ± 0.012 a	0.705 ± 0.029 b	0.960 ± 0.068 b	1.375 ± 0.013 a	2.009 ± 0.057 b	2.589 ± 0.005 b	3.285 ± 0.130 b	3.814 ± 0.037 b
1600	0.216 ± 0.025 a	0.449 ± 0.026 a	0.711 ± 0.031 b	0.964 ± 0.065 b	1.374 ± 0.015 a	1.919 ± 0.032 a	2.603 ± 0.113 b	3.286 ± 0.032 b	3.635 ± 0.077 a
3200	0.218 ± 0.023 a	0.431 ± 0.014 a	0.672 ± 0.038 b	0.953 ± 0.011 b	1.354 ± 0.010 a	1.869 ± 0.048 a	2.419 ± 0.069 a	3.226 ± 0.082 b	3.456 ± 0.336 a

### Effects of AgNPs on Silkworm Survival Rates and Cocoon Shell Weights

**Fig. 3:**
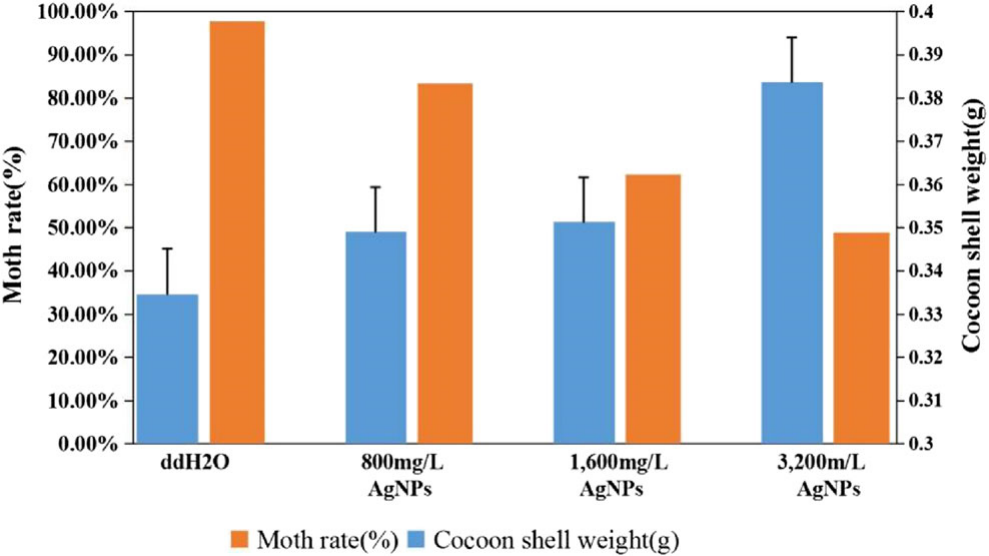
Effects of high concentrations of AgNPs on the cocoon shell weights and moth rates. With increasing concentrations of AgNPs, the cocoon shell weights of the silkworms showed an increase tendency, while moth rates gradually decreased. Statistical significance for all tests was judged at a probability level of 0.05 (*P* < 0.05)

AgNPs have no lethal effects on silkworm larvae at low concentrations (≤400 mg/L). The survival rates and cocoon shell weights of the silkworms were analyzed at ≥800 mg/L AgNP concentrations (Table 3 and Fig. 3). The results indicated that the larvae began to die when treated with 800 mg/L AgNPs, and they exhibited increased weight of the cocoon shells and significantly decreased moth rate. At ≥800 mg/L AgNP concentrations, the larval survival rates, cocoon shell weights, and the moth rates decreased remarkably, but the weights of the cocoon shells increased. At 3200 mg/L AgNP concentrations, the weight of the cocoon shells increased, but the moth rate was only 50 ± 10%. The results showed that high concentration (≥800 mg/L) of AgNPs increased weight of the cocoon shells of silkworms, which resulted in larval death.

**Table 3:** Effects of different concentrations of AgNPs on silkworm survival rate and cocoon shell weights

AgNPs (mg/L)	Silkworm^a^	Dia^a^	Cocoon^a^	Cocoon shells weight (g)^a^	Dead cocoon^a^	Moth^a^	Moth rate^a^ (%)
0	30	0	30	0.3347 ± 0.001 a	1	29	96.67
0	30	0	30	0.3355 ± 0.001 a	0	30	100.00
0	30	0	30	0.3339 ± 0.001 a	1	29	96.67
800	30	1	29	0.349 ± 0.001 b	5	24	80.00
800	30	0	30	0.3486 ± 0.001 b	4	26	86.67
800	30	1	29	0.3494 ± 0.001 b	4	25	83.33
1600	30	6	24	0.3513 ± 0.001 b	6	18	60.00
1600	30	7	23	0.3519 ± 0.001 b	7	18	60.00
1600	30	5	25	0.3507 ± 0.001 b	5	20	66.67
3200	30	9	21	0.3836 ± 0.001 b	6	15	50.00
3200	30	11	19	0.3842 ± 0.001 b	7	12	40.00
3200	30	7	23	0.3829 ± 0.001 b	6	17	56.67

^a^Results are expressed as mean ± SD

### Effects of AgNPs on the Fat Body Proteome in Silkworms

**Fig. 4:**
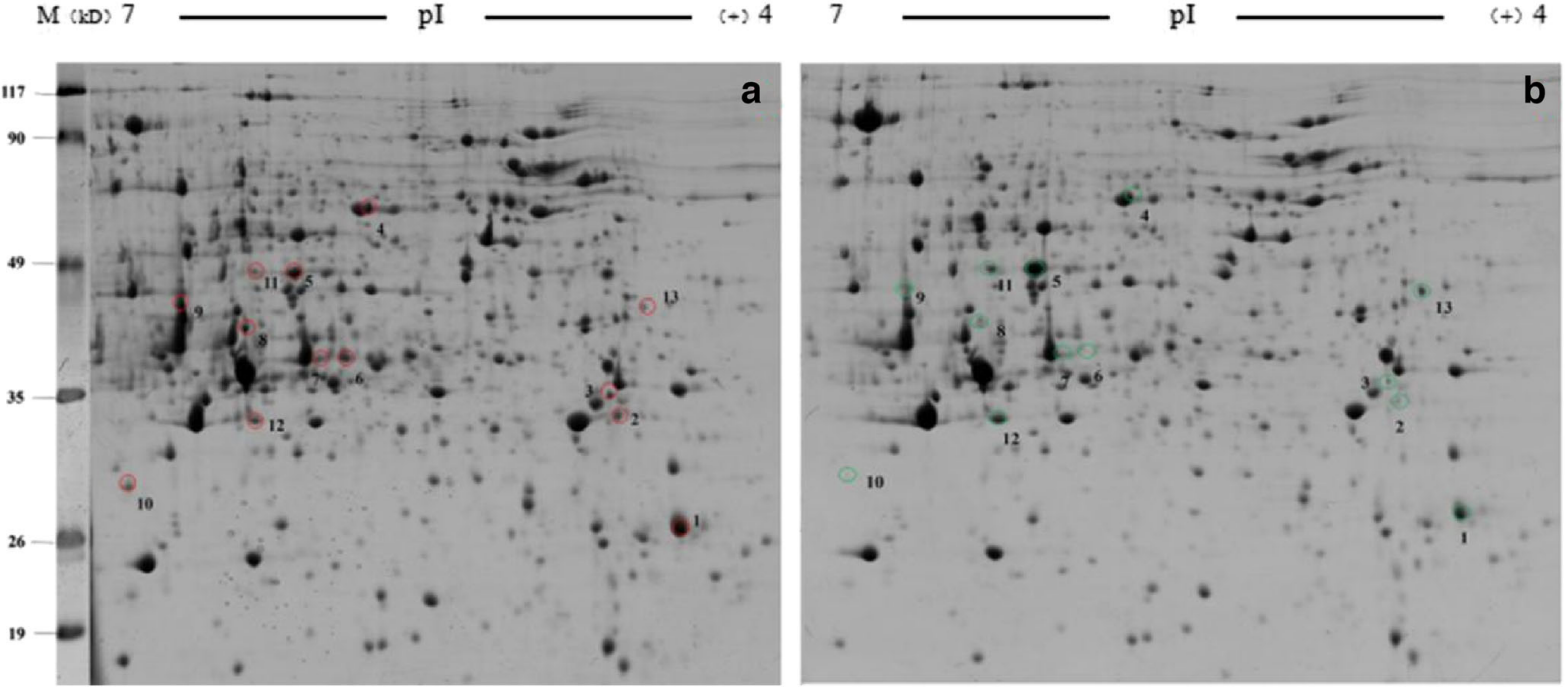
Two-dimensional electrophoresis results of fat body proteins. **a** The control group treated with ddH_2_O. **b** The group treated with 1600 mg/L AgNPs. *Numbered spots* represent differentially expressed proteins

As shown in Fig. 4, the software analysis showed that there were 13 significant differences between the fat body protein spots of the control group and the treatment group (1600 mg/L AgNPs). Eleven proteins were expressed in both groups, and two proteins were only expressed in the control group (Table 4).

**Table 4:** Identification of differentially regulated proteins in the control (ddH_2_O) and treatment (1600 mg/L AgNPs) groups

Spot no.^a^	Protein ID	Name	Gene name	Theoretical MW^b^ (kDa)/pI^c^		ANOVA *P* value	Fold change^d^	Express^e^
1	gi|512908327	Calexcitin-2-like	/	23.56	5.09	2.09E−04	2.03	↓
2	gi|13195043	Fibroin light chain, partial	*Fib-l*	24.83	4.53	0.00108697	2.28	↓
3	gi|512931543	Ubiquitin carboxyl-terminal hydrolase	/	25.1	5.02	2.82E−04	2.01	↓
4	gi|512915932	Cytosolic non-specific dipeptidase	/	58.83	6.15	6.94E−05	2.27	↓
5	gi|112983926	Arginine kinase	*AK*	32.50	7.23	1.36E−05	0.43	↑
6	gi|512915980	S-formylglutathione hydrolase	/	32.13	5.65	0.00128389	5.25	↓
7	gi|827538302	Low molecular 30 kDa lipoproteinPBMHP-12	*Lp-c12*	21.83	8.61	0.0338263	6.38	↓
8	gi|225905552	Low molecular lipoprotein 30K pBmHPC-6	*Lp-c6*	29.82	5.92	0.0464862	2.73	↓
9	gi|87248167	Isocitrate dehydrogenase, partial	/	46.55	6.24	0.00947802	11.25	↓
10	gi|112983420	Heat shock protein hsp 19.9	*Hsp19.9*	19.94	6.53	2.40E−04	2.67	↓
11	gi|827541166	Arginine kinase	*AK*	40.31	5.87	3.57E−04	0.27	↑
12	gi|112983028	Glutathione S-transferase sigma 1	*GSTs1*	23.60	5.98	0.00239861	0.48	↑
13	gi|6016405	Juvenile hormone-binding protein	*JHBP*	2.15	6.02	2.89E−05	0.44	↑

^a^Numbers indicate regions that were excised from the SDS-polyacrylamide gels for the mass spectrometry analysis

^b^Molecular weight

^c^Isoelectric point

^d^Fold change = control/treatment

^e^Upregulated expression “↑”; downregulated expression“↓”

### qPCR for Differentially Expressed Protein Validation

**Fig. 5:**
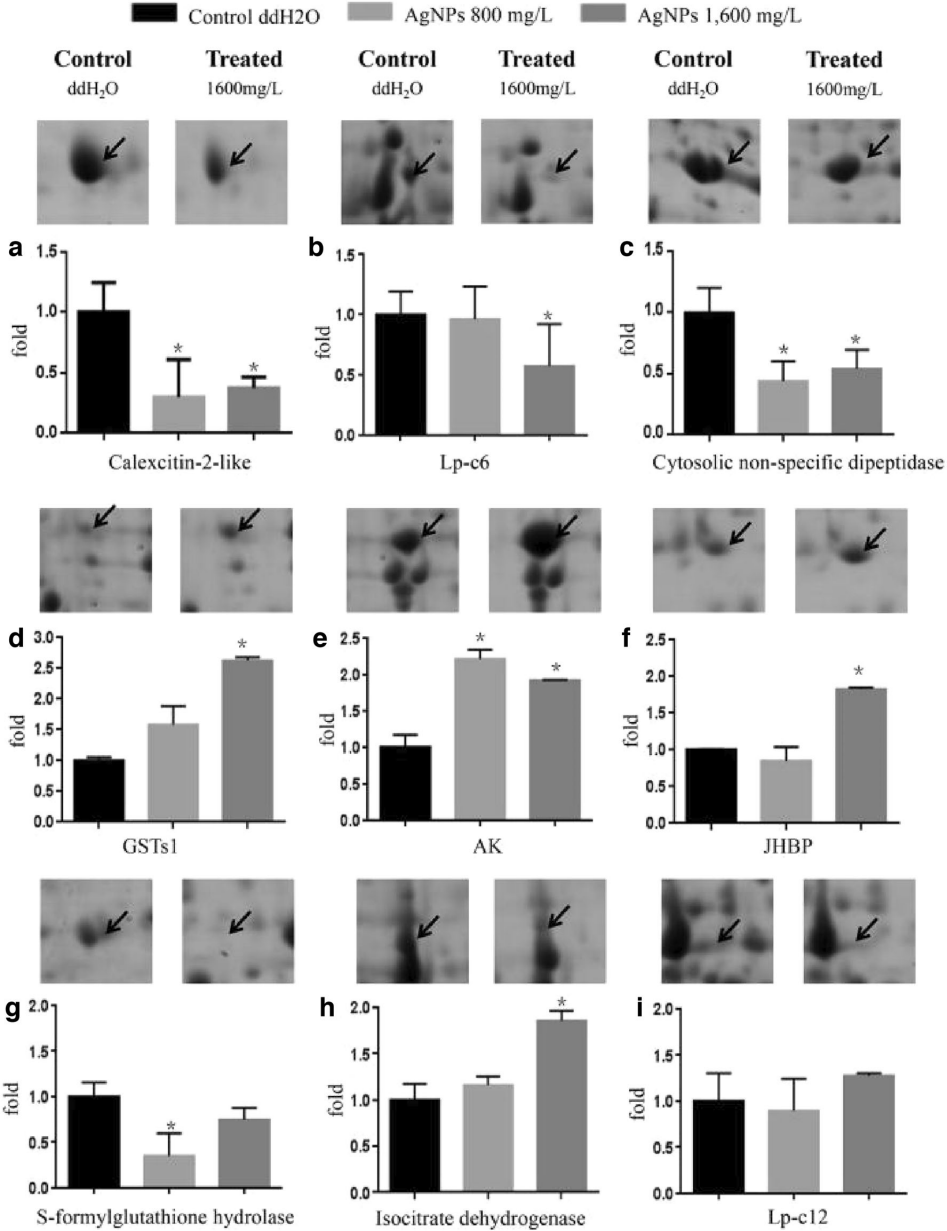
Differential expressed proteins and the expression of their corresponding genes as measured by qPCR. *Arrows* indicate significantly differentially expressed proteins. The results of the qPCR for genes in the control group are shown in *black*, while those of the 800 and 1600 mg/L AgNP groups are shown in *light gray* and *dark gray*, respectively. The experiments were repeated three times, and statistically significant differences (mean ± SD, *P* < 0.05) are indicated

cDNA was isolated from fifth-instar larvae and used as template after the larvae were fed AgNPs for 144 h. qPCR was performed to examine the expression of genes encoding the fat body proteins that were differentially expressed between the control group and the AgNP groups (800 and 1600 mg/L) (Fig. 5). Compared with the control group, the calexcitin-2-like (A) and cytosolic non-specific dipeptidase (C) genes were downregulated significantly, and glutathione S-transferase s1 (GSTs1) (D) genes and *AK* (E) were upregulated significantly in the treatment groups, which is consistent with the corresponding protein expression levels. The expression of the *LP-C6* (B) did not differ significantly between the 800 and 1600 mg/L AgNP groups. The expression of the S-formylglutathione hydrolase gene (G) was downregulated significantly in the 800 mg/L AgNP group, and it was downregulated slightly in the 1600 mg/L AgNP group. The expression of the genes encoding juvenile hormone binding protein (JHBP) (F) and isocitrate dehydrogenase (H) was not significantly changed in the 800 mg/L AgNP group, but it was upregulated significantly in the 1600 mg/L AgNP group. The expression of the gene encoding *LP-C12* (I) did not differ significantly between the control and treatment groups. The expression levels of seven genes were consistent with those of their corresponding protein spots following treatment with 1600 mg/L AgNPs. There were no differences in the expression levels of the genes encoding the other six protein spots (Fig. 5).

## Discussion

In the present study, we observed that AgNPs promoted the growth of silkworms and induced their death. To determine the action mechanism of AgNPs, we identified seven protein spots that were differentially expressed following treatment with 1600 mg/L AgNPs. Furthermore, a functional analysis of the significantly differentially expressed proteins indicated that AK is a phosphokinase that plays critical roles in the metabolism, storage, and utilization of energy in invertebrates [30]. In addition, AK may play an important role in the insect immune response and environmental adaptation. Kang showed that the expression of AK in the midgut of NB and BC8 larvae, which are resistant to *B. mori* nuclear polyhedrosis virus, is higher than that of 306 larvae, indicating that AK protects silkworm larvae against viral infection [31]. GSTs1 is a multifunctional enzyme in vivo, and it plays major roles in protecting against oxidative damage, as well as in antioxidant processes, detoxification, and metabolism, including GSH-independent peroxidase activity [32, 33]. A study found that the mercapto group of biological systems may be involved in the transport of AgNPs [34]. GST plays an important role in the detoxification of insecticides [35]. The expressions of AK and GSTs1 were upregulated after silkworms being fed AgNPs. In the present study, this may be related to an emergency response that induced toxic effects and immune responses in the presence of high concentrations of AgNPs in silkworms.

LP-c is a low-molecular-weight (30 kDa) protein that is synthesized in the fat body. It is an important storage protein during silkworm growth and development. It was shown that LP-c could bind to the ecdysone receptor-B1 (EcR-B1) and thus inhibit the binding of EcR-B1 to ultraspiracle (USP), leading to the failure of EcR-B1 in activating the expression of downstream genes, thereby inhibiting apoptosis [36]. This 30 kDa protein can prolong life and inhibit programmed cell death in insects [37]. Heat shock protein 19.9 (HSP 19.9) is a member of the small HSP family, which plays an important role in protecting cells from heat-induced damage [38]. It is also involved in the protection against heat stress-induced apoptosis and other phenomena [39]. These results are similar to those obtained in *Drosophila melanogaster* [40]. HSPs induce cell growth and differentiation in the presence of oxidative stress in mammalian cells [41]. The expressions of LP-c and HSP 19.9 were downregulated after silkworms being treated with AgNPs. The results showed that when the concentration of AgNPs reached 1600 mg/L, the expression of the LP-c protein was altered, and the apoptosis and death of silkworm cells appeared. Cytosolic non-specific dipeptidase 2 (CNDP2) is a dipeptide metalloproteinase that catalyzes the cleavage of dipeptide B-alanyl-l-histidine [42]. The CNDP2 gene encodes a non-specific carnosinase that has a high affinity for Cys-Gly in the γ-glutamyl cycle, and it is involved in the biosynthesis of GSH [43]. GSH acts as a detoxification agent in the body. Thus, the downregulated expression of the CNDP2 protein will decrease GSH synthesis, and detoxification, and result in the death of silkworms.

Calexcitin, a signaling protein that binds calcium and GTP, inhibits potassium channels. Calexcitin, which contains an EF-hand domain pair, is involved in binding to metal ions and increasing the diversity of the regulation of calcium-binding proteins [44]. After silkworms were fed AgNPs, the expression of calexcitin was downregulated, which affects the cell membrane potential, nerve conduction, and the signal pathways of silkworms.

Low-molecular-weight JHBPs are specific vectors for juvenile hormone (JH) in the hemolymph of butterflies and moths. As hormone signal transporter, JHBPs have a profound impact on the growth and development of insects [45]. Previous reports suggested that JH binds to three types of JHBPs, including lipoproteins, hexamers, and low-molecular-weight proteins of approximately 30 kDa [46–48]. Adding JH to larvae can extend the period of eating mulberry leaves, the synthesis of fibroin, and the weights of the cocoon shells [49]. In our study, the period of eating mulberry leaves was extended. Therefore, we speculate that the increase weights of the cocoon shells may be related to the upregulation of expression of JHBPs after treatment with AgNPs. It may also be associated with the upregulation of cytosolic non-specific dipeptidase and AK protein expression, which results in the increased storage and utilization of carbohydrates in silkworms. Previous research also showed that AgNPs exhibit the presence of certain growth stimulant activity and can increase the silk yield [50]. Moreover, the results of a Kyoto Encyclopedia of Genes and Genomes analysis showed that isocitrate dehydrogenase and S-formylglutathione hydrolase, the key rate-limiting enzymes in the carbon cycling pathway, were both downregulated after the addition of AgNPs, which results in the slower use of carbohydrates by fat bodies, as well as associated metabolic changes.

**Fig. 6:**
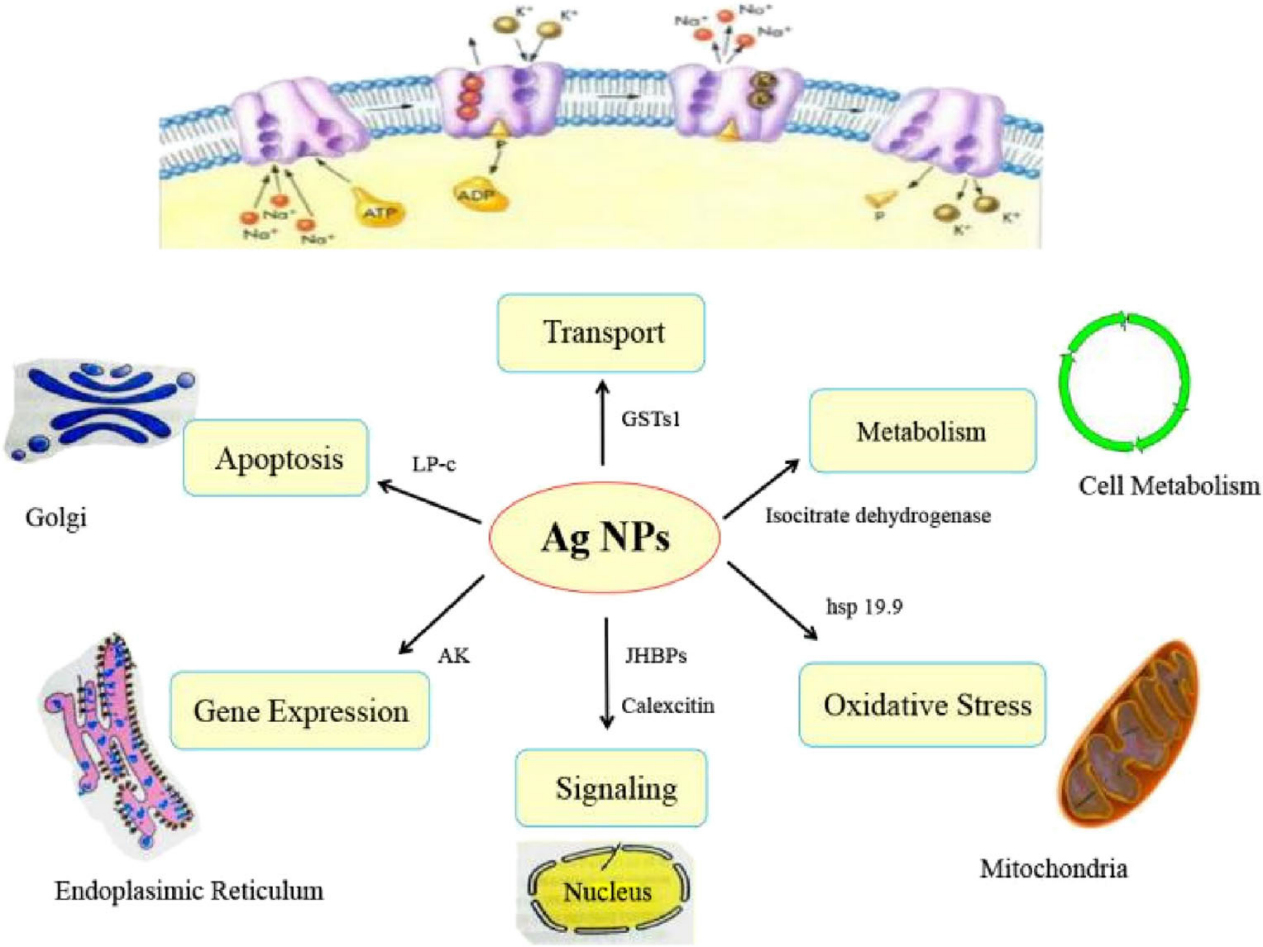
The biological consequences of silkworm exposure to AgNPs

In the present study, growth-inhibiting and toxic effects of AgNPs on silkworms were observed at the individual level. We found that AgNPs influenced the functions of the metabolic cycle, as well as signal transduction, apoptosis, and ion transport (Fig. 6). AgNPs could influence carbon regulatory proteins during metabolism, thereby weakening their metabolic function and increasing energy storage and utilization. AgNPs also can reduce the ability of silkworms to withstand oxidative stress, interfere with programmed cell death, and attenuate the expression of detoxification proteins. Overall, AgNPs have large potential toxic effects on human health and the environment; therefore, they should be used with caution.
